# Bionematicides as an alternative to methyl bromide fumigation

**DOI:** 10.21307/jofnem-2021-044

**Published:** 2021-04-12

**Authors:** B. B. Westerdahl, J. Hasey, J. Grant, L. W. Beem

**Affiliations:** 1 Department of Entomology and Nematology, University of California, Davis, CA 95616; 2 University of California Cooperative Extension, Yuba City, CA 95991; 3 University of California Cooperative Extension, Stockton, CA 95206; 4 Beem Consulting, Sacramento, CA 95827

**Keywords:** Biological Control, DiTera, Management, *Mesocriconema Xenoplax*, *Myrothecium Verrucaria*, Nema-Q, Nematode, *Pratylenchus Vulnus*, *Quillaja Saponaria*, Walnut

## Abstract

Fumigant use in perennial crops can be reduced through prolonging the life of existing orchards. The longer an orchard remains healthy and productive, the less often it will be terminated, fumigated, and replanted. Two trials were conducted to determine the effectiveness of DiTera, a toxin produced by the fungus (*Myrothecium verrucaria)* and Nema-Q, an extract of the soap bark tree (*Quillaja saponaria*) for management of root-lesion (*Pratylenchus vulnus*) and ring (*Mesocriconema xenopla)* nematodes on walnuts. In the first trial, spring and fall treatments of DiTera were applied each year for four years to variety ‘Chandler’ scion on seedling ‘Paradox’ rootstock, and to own-rooted ‘Chandler’ trees. On ‘Paradox’ rootstock, both DiTera and Nema-Q increased walnut yields (*P* ≤ 0.05) and produced more vigorous trees (*P* ≤ 0.05) without significant reductions in nematode populations (*P* ≤ 0.05). A second trial was conducted with three rates of DiTera and four rates of Nema-Q, combinations of the two products, and an untreated control on ‘Chandler’ scion on ‘Paradox’ rootstock. The highest rate of Nema-Q (*P* ≤ 0.05), and a combination treatment of DiTera plus Nema-Q (*P* ≤ 0.05) increased trunk circumference. Several treatments showed reductions in root-lesion and ring nematodes (*P* ≤ 0.05). Bionematicides can improve yield, growth, and vigor in walnut orchards infested with plant-parasitic nematodes.

Root-lesion, *Pratylenchus vulnus* Allen and Jensen; and ring, *Mesocriconema xenoplax* (Raski, 1952) Loof & De Grisse, 1989 nematodes reduce walnut (*Juglans* sp.) yields through root damage from direct feeding and by placing trees under stress (Lownsbery, 1956, 1959; Lownsbery et al., 1978). Root-lesion nematodes are likely to be found within roots as well as in soil, while ring nematodes are external parasites of roots. Recently, two biological nematicides achieved registration in California for use on walnuts, DiTera, a toxin produced by the fungus *Myrothecium verrucaria* (Alb. & Schwein.) Ditmar (1813) and Nema-Q, an extract of the soap bark tree *Quillaja saponaria* Molina.

In recent years, extensive research has been conducted to find replacements for methyl bromide, widely used as a pre-plant soil fumigant before being implicated in the depletion of ozone in the stratosphere (United States Environmental Protection Agency, 1993). A way to reduce the frequency of fumigant use in perennial crops is through prolonging the life of existing orchards. The longer an orchard remains healthy and productive, the less often it will be terminated, fumigated, and replanted. Over time, this reduces the amount of fumigant used. In addition to fumigation, a variety of approaches have been researched for management of plant-parasitic nematodes including development of pre-plant hot water treatments of rootstocks, evaluation of rootstock susceptibility, and evaluation of biological products (Buzo et al., 2009; Giraud et al., 2011; Hasey et al., 2004; Westerdahl and Radewald, 2011).

Only about a dozen nematicidal active ingredients have ever achieved registration in California, and several of the most effective of these lost their registrations owing to groundwater contamination, air pollution, or carcinogenicity (Ferris, 2021). The loss of use of the nematicide dibromochloropropane (DBCP) in 1977, that had been widely used post-plant on bearing fruit and nut tree crops in California, created a tremendous need for replacement products (United States Environmental Protection Agency, 2014).

The goal of this study was to evaluate the potential of DiTera and Nema-Q for post-plant management of root-lesion and ring nematodes in commercial walnut orchards.

## Materials and methods

Two field trials were conducted to evaluate the potential of two bionematicides for nematode management on walnuts. Because the actual active ingredients in both products tested are uncertain, all rates are expressed in terms of amount of product per ha.

Sutter County Trial: The first trial was conducted in an orchard in Sutter County, CA on Holillipah loamy sand. This orchard was previously used for an own-rooted ‘Chandler’ compared to ‘Chandler’ on ‘Paradox’ rootstock trial (Hasey et al., 2004). In that trial that was planted in 1991, two rootstocks, micropropagated ‘Chandler’ (*Juglans regia* L.) on its own-roots and nursery grafted ‘Chandler’ on seedling ‘Paradox,’ *J. hindsii* (Jeps.) Jeps. Ex R.E. Sm., *x J. regia*, rootstock were spaced at 7.62 m  × 7.62 m in a randomized complete block design with 20 individual tree replicates. In 1998, the orchard was found to be infested with plant-parasitic nematodes. From this previous trial, 15 trees of each rootstock were selected for a trial in a randomized complete block design with five replicates of three treatments: Untreated Control (UC), DiTera (DT), and Nema-Q (NQ). DiTera^®^ DF (Valent BioSciences Corporation, Libertyville, IL) at 56 kg/ha, and Nema-Q^®^ (Monterey AgResources, Fresno, CA) at 23.4 L/ha, were applied in a 50% banded spray, so that actual amounts applied were 28 kg DT and 11.7 L NQ/treated ha. One day prior to treatment, 1.25 cm of irrigation was applied to the orchard from 39.8 Lph microsprinklers (Antelco Rotor Spray Mini Sprinkler, Antelco, Longwood, FL). Each tree was treated individually with 5.7 L of solution from a 7.6-L watering can (Bloem Classic 2 Gal. Blue Plastic Watering Can, Bloem, Hudsonville, MI) twice yearly for four years. Treatment was immediately followed by an additional 1.25 cm of irrigation. Treatments were applied in 2003 (April 11 and October 24), 2004 (April 6 and September 29), 2005 (April 11 and October 13), and 2006 (May 10 and October 30). The orchard was irrigated by the grower as needed following California irrigation Management Information System (CIMIS) guidelines (CIMIS station #84) (CIMIS, 2021). Weather data for the trial area is available from CIMIS station #84 (UCIPM, 2021). The orchard was managed by the grower and treated with standard practices with respect to fertilization, insecticides, and fungicides.

Soil samples were taken prior to each treatment date using a 5-cm diameter bucket auger to a depth of 60-cm midway between the dripline of the tree canopy and the tree trunk, in the fall and spring of each year. Nematodes were extracted from a 400-cm^3^ soil sub-sample with a modified semiautomatic elutriator and sucrose centrifugation technique (Byrd et al., 1976). Extracted nematodes were identified and counted at × 45 magnification under a stereoscopic dissecting microscope (Bausch & Lomb, Bridgewater, NJ). The sampling method used did not yield sufficient roots to permit extraction of nematodes. Yields and trunk circumference were evaluated each year from 2002 (pretreatment baseline) to 2006. Circumference of each tree trunk was measured at 60 cm above the ground and trunk cross-sectional area was calculated from the circumference measurements (Retzlaff et al., 1992). In addition, tree vigor was visually evaluated in 2005 and 2006 using a rating scale developed by the authors: 0=Dead, 1=Very low vigor, dieback, 2=Early yellowing, 3=No new shoot growth, 4=Some new shoot growth, and 5=Most vigorous.

San Joaquin County Trial: The second trial was conducted in San Joaquin County, CA with ‘Chandler’ scion on ‘Paradox’ rootstock. The randomized complete block trial with 6 replicates of 14 treatments evaluated three rates of DT, four rates of NQ and six combinations of DT and NQ for their effectiveness in controlling root-lesion and ring nematodes. Treatments included an UC. Treatments were applied to the soil surface in a 50% banded spray. One day prior to treatment, 1.25 cm of irrigation was applied to the orchard with 30.2 Lph microsprinklers (Rain Bird Micro-Quick Spray Assembly, Rain Bird, Azusa, CA). Each tree was treated individually with 5.7  L of solution from a 7.6-liter watering can (Bloem Classic 2 Gal. Blue Plastic Watering Can, Bloem, Hudsonville, MI). Treatment was immediately followed by an additional 1.25 cm of irrigation. Orchards were irrigated by the grower as needed following CIMIS guidelines (CIMIS station #70) (CIMIS, 2021). Weather data for the trial area is available from CIMIS station #70 (UCIPM, 2021). The orchard was managed by the grower and treated with standard practices with respect to fertilization, insecticides, and fungicides.

Treatment effectiveness was evaluated via soil and root sampling, and trunk circumference measurements. Nematodes were extracted from soil as described for the Sutter County Trial. Nematodes were also extracted from roots that were weighed and placed in an intermittent mist chamber for 72 h (Ayoub, 1977). Treatments were applied in the spring of 2005 following measurement of trunk circumference. Additional nematode sampling followed by repeated treatments were conducted in October 2005, April 2006, and October 2006. Post-treatment trunk circumference measurements were done in October 2005, April 2006, and October 2006.

Data analysis: Both trials were conducted in randomized complete block design. There were five replicates per treatment in the Sutter County Trial and data were analyzed using Repeated Measures Analysis of Variance (ANOVA) (*P* ≤ 0.05), followed by independent contrasts for mean separation (*P* ≤ 0.05) (Super Anova, Abacus Concepts, Berkeley, CA); and by linear regression and correlation analysis (*P* ≤ 0.05) (Prism 7, GraphPad Software, Inc., San Diego, CA). There were six replicates per treatment in the San Joaquin County Trial and data were analyzed with Repeated Measures Analysis of Variance ANOVA (*P* ≤ 0.05), followed by independent contrasts for mean separation (*P* ≤ 0.05) (SuperAnova, Abacus Concepts, Berkeley, CA).

## Results

Sutter County Trial: Pretreatment yield data taken in 2002 indicated that there were no significant differences at the beginning of the Sutter County trial (*P* > 0.05) (Table 1). During the course of the trial, combined data for ‘Paradox’ and own-rooted treatments indicated yields for the trees treated with NQ treatment were greater than for UC trees in 2003 (P ≤ 0.0027), 2006 (*P* ≤ 0.0041), and 2007 (*P* ≤ 0.0080). For trees treated with DT, yields were greater than for UC trees in 2006 (*P* ≤ 0.0068) and 2007 (*P* ≤ 0.0004). For ‘Paradox’ rootstock, yields for trees treated with NQ were greater than UC trees for 2003 (*P* ≤ 0.0338), 2005 (*P* ≤ 0.0008), 2006 (*P* ≤ 0.0031), and 2007 (*P* ≤ 0.0279). Yields for trees treated with DT were greater than UC trees for 2005 (*P* ≤ 0.0325) and 2006 (*P* ≤ 0.0114). For own-rooted trees, yields for trees treated with NQ were greater than UC trees in 2003 (*P* ≤ 0.0383), as were yields for trees treated with DT in 2007 (*P* ≤ 0.0005). Overall, from 2002 to 2007, yields for UC trees did not increase. In contrast, compared to UC trees, yields increased for trees treated with DT (*P* ≤ 0.0142) or with NQ (*P* ≤ 0.0013) on ‘Paradox’; for trees treated with DT (*P* ≤ 0.0001) or with NQ (*P* ≤ 0.0494) on own-rooted; and for trees treated with DT (*P* ≤ 0.0001) or with NQ (*P* ≤ 0.0003) on both rootstocks combined.

**Table 1.** Yield data for Sutter County trial.

		Yield (kg/tree)					
Year	Treatment	Combined		Paradox		Own-rooted	
2002	UC	23.4		26.3		20.5	
	DT	24.0		25.2		22.8	
	NQ	28.8		32.4		25.0	
2003	UC	32.0		37.4		26.5	
	DT	36.9		40.8		32.9	
	NQ	42.1	0.0027^a^	47.0	0.0338	37.1	0.0383
2004	UC	22.8		25.45		20.2	
	DT	27.1		31.1		23.0	
	NQ	26.5		33.1		20.0	
2005	UC	33.0		33.8		32.2	
	DT	39.3		43.4	0.0325	35.3	
	NQ	38.3		49.2	0.0008	27.4	
2006	UC	33.0		37.7		28.3	
	DT	42.3	0.0068	49.1	0.0114	35.5	
	NQ	42.6	0.0041	51.2	0.0031	33.9	
2007	UC	27.0		31.2		22.8	
	DT	39.4	0.0004	37.4		41.4	0.0005
	NQ	35.8	0.0080	41.1	0.0279	30.6	
Yield difference (kg/tree) between UC in 2002, and UC, DT, and NQ in 2007							
	UC	3.6		4.9		2.3	
	DT	16.0	0.0001	11.1	0.0142	20.9	0.0001
	NQ	12.5	0.0003	14.9	0.0013	10.1	0.0494

Notes: Data are means of 5 replicates.

a Figures indicate the probability of that treatment being different from the UC.

Pretreatment, in 2002, there were no differences in trunk cross sectional area for both rootstocks combined or for own-rooted trees (*P* > 0.05) (Table 2). For ‘Paradox,’ initial trunk cross sectional area for trees treated with NQ was greater than that for UC trees (*P* ≤ 0.0396). For both rootstocks combined, trunk cross sectional area for trees treated with NQ was greater than UC trees for 2003 (*P* ≤ 0.0064), 2004 (*P* ≤ 0.0009), 2005 (*P* ≤ 0.0012), 2006 (*P* ≤ 0.0001), and 2007 (*P* ≤ 0.0001), and for trees treated with DT in 2006 (*P* ≤ *0.0428*). For ‘Paradox’ rootstock, trunk cross sectional area was greater than the UC trees for trees treated with DT in 2003 (*P* ≤ 0.0396), 2004 (*P* ≤ 0.0302), 2005 (*P* ≤ 0.0420), 2006 (*P* ≤ 0.0044), and 2007 (*P* ≤ 0.0037), and for trees treated with NQ in 2003 (*P* ≤ 0.0001), 2004 (*P* ≤ 0.0001), 2005 (*P* ≤ 0.0001), 2006 (*P* ≤ 0.0001), and 2007 (*P* ≤ 0.0001). For own-rooted trees, trunk cross sectional area of trees treated with DT was greater than UC trees in 2003 (*P* ≤ 0.0003). Overall, differences in trunk cross sectional area between when the trial was initiated in 2002 and terminated in 2007 were greater (*P* ≤ 0.05) for all rootstock and treatment combinations (data not shown).

**Table 2.** Trunk cross-sectional area for Sutter County trial.

		Trunk cross-sectional area (cm)					
Year	Treatment	Combined		Paradox		Own-rooted	
2002	UC	354		221		488	
	DT	352		279		425	
	NQ	386		324	0.0396	448	
2003	UC	422		292		552	
	DT	396		363	0.0396	428	0.0003
	NQ	483	0.0064^a^	449	0.0001	516	
2004	UC	448		324		572	
	DT	468		400	0.0302	535	
	NQ	526	0.0009	509	0.0001	542	
2005	UC	460		354		567	
	DT	476		425	0.0420	526	
	NQ	535	0.0012	542	0.0001	528	
2006	UC	503		386		620	
	DT	555	0.0428	487	0.0044	623	
	NQ	605	0.0001	613	0.0001	597	
2007	UC	533		421		647	
	DT	571		525	0.0037	619	
	NQ	648	0.0001	677	0.0001	620	

Notes: Data are means of 5 replicates.

a Figures indicate the probability of that treatment being different from the UC.

Visual rating of tree vigor conducted in 2005 indicated ‘Paradox’ trees treated with NQ were more vigorous than UC trees (*P* ≤ 0.02) (Table 3). ‘Paradox’ trees treated with DT were more vigorous than UC trees in 2006 (*P* ≤ 0.04). Own-rooted trees treated with NQ were more vigorous than UC trees in 2005 (*P* ≤ 0.04).

**Table 3.** Tree rating ranging from 0 (dead) to 5 (most vigorous) for Sutter County trial^a^.

Year	Treatment	___Paradox____		_Ownroot___	
2005	UC	3.6		3.0	
	DT	3.8		3.6	
	NQ	4.4	0.02^b^	3.8	0.04
2006	UC	3.9		3.5	
	DT	4.6	0.04	4.2	
	NQ	4.6		4.1	

Notes: Data are means of 5 replicates.

a Rating Scale: 0=Dead, 1= Very low vigor, dieback, 2=Early yellowing, 3=No new shoot growth, 4=Some new shoot growth, 5=Most vigorous.

b Figures indicate the probability of that treatment being different from the UC.

Regression and correlation analysis support the results discussed above. Slopes of the lines are not significantly different, but the y intercepts for yield are significantly different between trees treated with DT and UC trees. Linear regression analysis over time demonstrated positive but not significant slopes for yield (*P* > 0.05) for all treatments except for own-root UC trees that had a negative slope. The elevation of the line for own-root trees treated with DT (*yield =* 0.6822 *+* 6.8978*time) was greater (*P* ≤ 0.025) than that for UC trees (*yield = −*29.5084−1.5548*time).

For trunk cross sectional area (TCSA), linear regression demonstrated positive slopes for all treatments that were significant at *P* ≤ 0.05. For trees treated with NQ, y intercepts were significantly different from UC trees for both ‘Paradox’ and own-rooted trees. Line elevation for own-rooted trees treated with NQ (*TCSA =* 9.8716 + 3.8494*time, *P* ≤ 0.0158) was greater than that for UC trees (*TCSA =* 7.3885 + 2.9901*time). The same relationship was true for trees treated with NQ that were on ‘Paradox’ rootstock (*TCSA =* 23.0473 + 5.9105*time, *P* ≤ 0.0009) compared to UC trees (*TCSA =* 20.221 + 5.7692*time).

Prior to treatment on the first sampling date, there were no differences in numbers of root-lesion or ring nematode for either rootstock (*P* > 0.05) (Table 4). Throughout the course of the trial, reductions were not observed in nematode populations in soil (*P* > 0.05). For trees treated with DT, increases in the population of ring nematode in soil were observed on ‘Paradox’ in October of 2006 (*P* ≤ 0.050), and on own-rooted trees in April 2004 (*P* ≤ 0.004), October 2004 (*P* ≤ 0.002), and April 2005 (*P* ≤ 0.04). An increase in the population of root-lesion nematode for trees treated with NQ was observed on own-rooted trees in October 2005 (*P* ≤ 0.006).

**Table 4.** Effect of treatments on densities of nematodes per 1,000 cm^3^ soil in Sutter County Trial.

Sample		______Paradox_____			___________Own-rooted____			
Date	Treatment	Lesion	Ring		Lesion		Ring	
October 2003	UC	3,030	0		4,380		10	
	DT	3,200	0		5,740		100	
	NQ	3,820	0		4,410		0	
April 2004	UC	1,440	40		2,560		110	
	DT	1,330	540		2,020		3,270	0.004
	NQ	1,320	300		1,870		0	
October 2004	UC	1,130	20		1,230		450	
	DT	2,250	30		2,470		3,830	0.002
	NQ	1,210	420		1,580		0	
April 2005	UC	870	40		610		300	
	DT	1,410	0		580		2,480	0.040
	NQ	2,290	260		1,350		10	
October 2005	UC	1,470	10		1,890		1,380	
	DT	2,060	30		2,580		2,340	
	NQ	2,850	540		4,970	0.006	30	
April 2006	UC	1,380	1,610		1,640		590	
	DT	2,650	980		1,300		2,560	
	NQ	1,310	2,020		1,680		830	
October 2006	UC	470	280		740		470	
	DT	320	1,340	0.050^a^	960		290	
	NQ	620	1,030		2,080		660	

Notes: Data are means of 5 replicates.

a Figures indicate the probability of that treatment being different from the UC.

San Joaquin County Trial: In the San Joaquin County trial, in October 2005, six months after treatment, numerically, 10 out of 13 treatments showed an increase in trunk circumference ranging from 7.13 to 12.05% compared to a 7.1% increase for the UC trees (Table 5). The largest increases were 12.05% for trees treated with NQ at 56 L/ha (*P* ≤ 0.0105) followed by 11.36% for trees treated with DT 28 kg/ha + NQ 37 L/ha (*P* ≤ 0.0269). These same two treatments continued to show significant increases in trunk circumference on subsequent sampling dates (*P* ≤ 0.05). Trees treated with DT at 14 kg/ha was the only treatment that failed to show a numerical increase in trunk circumference compared to UC on at least one occasion (*P* > 0.05).

**Table 5.** Circumference of trees in San Joaquin County trial.

Treatment	Initial trunk	Percent increase from initial circumference					
(L/ha)	Circumference (cm)	October 2005		April 2006		October 2006	
UC	68.3	7.1		7.2		10.6	
DT 56	66.5	7.0		7.7		10.6	
DT 28	61.4	7.2		8.0		10.9	
DT 14	67.1	5.3		5.7		7.9	
DT 56 + NQ 37.4	71.2	8.4		8.9		11.7	
DT 56 + NQ 23.4	69.7	9.2		9.9		12.8	
DT 28 + NQ 37.4	61.5	11.4	0.0269^a^	12.9	0.0052	16.3	0.0091
DT 28 + NQ 23.4	68.2	8.5		9.5		12.8	
DT 14 + NQ 37.4	69.0	7.3		8.1		11.1	
DT 14 + NQ 23.4	71.7	7.1		5.9		10.0	
NQ 56	70.9	12.1	0.0105	12.8	0.0062	15.8	0.0176
NQ 37.4	64.0	7.6		8.6		11.3	
NQ 23.4	72.3	6.9		7.7		10.4	
NQ 12	61.6	7.2		8.1		10.8	

Notes: Data are means of 6 replicates.

a Figures indicate the probability of that treatment being different from the UC.

At the time of application in April 2005, although the number of root-lesion nematode in soil was numerically greater than the UC trees for all but one treatment, there were no significant differences between treatments (*P* > 0.05) (Table 6). When sampled in October 2005, trees treated with DT at 14 kg/ha + NQ 23.4 L/ha was the only treatment to show a reduction in the soil population of nematodes (*P* ≤ 0.0005). When sampled in October 2006, trees treated with DT at 28 kg/ha + NQ 23.4 L/ha (*P* ≤ 0.05) was the only treatment to show a reduction in nematodes in soil.

**Table 6.** Effect of treatments on densities of lesion nematodes per 1,000 cm^3^ soil in San Joaquin County trial.

Treatment	Date sampled				
(L/ha)	April 2005	October 2005		April 2006	
UC	2,783	4,208		2,950	
DT 56	6,708	4,167		2,375	
DT 28	4,825	8,075		6,075	
DT 14	4,458	4,883		3,875	
DT 56 + NQ 37.4	4,233	7,417		1,967	
DT 56 + NQ 23.4	3,708	6,625		2,350	
DT 28 + NQ 37.4	8,733	4,583		1,525	
DT 28 + NQ 23.4	3,492	5,325		1,575	0.0500
DT 14 + NQ 37.4	5,625	8,050		2,200	
DT 14 + NQ 23.4	8,405	2,333	0.0005^a^	3,150	
NQ 56	4,667	4,850		4,475	
NQ 37.4	2,817	6,600		2,650	
NQ 23.4	3,875	5,433		1,633	
NQ 12	2,517	3,175		1,583	

Notes: Data are means of 6 replicates.

a Figures indicate the probability of that treatment being different from the UC.

At the time of application in April 2005, roots from trees treated with DT at 14 kg/ha (*P* ≤ 0.02) and with NQ at 37.4 L/ha (*P* ≤ 0.05) had more root-lesion nematode per gram of root than the UC (Table 7). In October 2005, roots from trees treated with NQ at 12 L/ha had a greater number of root-lesion nematode per gram of root than UC trees (*P* ≤ 0.01). In April 2006, there were no differences in number of root-lesion nematode per gram of root.

**Table 7.** Effects on lesion nematode per gram of roots in San Joaquin County trial.

Treatment	Date sampled				
(L/ha)	April 2005	October 2005		April 2006	
UC	8		9		0
DT 56	142		9		2
DT 28	121		18		4
DT 14	203	0.02^a^	109		4
DT 56 + NQ 37.4	11		12		5
DT 56 + NQ 23.4	18		15		2
DT 28 + NQ 37.4	8		81		1
DT 28 + NQ 23.4	45		4		0
DT 14 + NQ 37.4	36		74		1
DT 14 + NQ 23.4	1		22		2
NQ 56	45		94		13
NQ 37.4	130	0.05	13		16
NQ 23.4	165		25		5
NQ 12	11		229	0.01	1

Notes: Data are means of 6 replicates.

a Figures indicate the probability of that treatment being different from the UC.

At the time of application in April 2005, there were no differences in number of ring nematode per liter of soil (*P* > 0.05) (Table 8). In October 2005, trees treated with DT at 14 kg/ha + NQ 23.4 L/ha and with NQ 56 L/ha had fewer ring nematode in soil than UC trees (*P* ≤ 0.01). In April 2006, trees in five treatments had fewer ring nematode in soil than UC: DT 56 kg/ha + NQ 23.4 L/ha (*P* ≤ 0.03), DT 28 kg/ha + NQ 37.4 L ha^−1^ (*P* ≤ 0.003), DT 14 kg/ha + NQ 23.4 L/ha (*P* ≤ 0.003), NQ 56 L/ha (*P* ≤0.003), and NQ 23.4 L/ha (*P* ≤ 0.03).

**Table 8:** Effect of treatments on densities of nematodes per 1,000 cm^3^ soil in San Joaquin County trial.

Treatment	Ring nematode				
(L/ha)	April 2005	October 2005		April 2006	
UC	975	317		1,575	
DT 56	1,133	7,300		2,150	
DT 28	1,500	950		525	
DT 14	83	1,117		900	
DT 56 + NQ 37.4	583	450		2,000	
DT 56 + NQ 23.4	3,283	2,500		350	0.03
DT 28 + NQ 37.4	83	300		0	0.003
DT 28 + NQ 23.4	900	1,650		1,600	
DT 14 + NQ 37.4	575	1,183		825	
DT 14 + NQ 23.4	63	0	0.01^a^	0	0.003
NQ 56	0	0	0.01	200	0.003
NQ 37.4	150	800		1,275	
NQ 23.4	750	4,883		475	0.03
NQ 12	2,900	950		1,575	

Notes: Data are means of 6 replicates.

a Figures indicate the probability of that treatment being different from the UC.

## Discussion

Bionematicides were evaluated on two walnut rootstocks, own-rooted ‘English’ (‘Chandler’) and ‘Paradox.’ Own-rooted ‘English’ walnut trees can be used in areas where commonly used rootstocks such as ‘Paradox’ (*J. hindsii and J. hindsii x J. regia*) are undesirable because of hypersensitivity to cherry leaf roll virus. This disease is known as walnut blackline because a black line forms at the graft union in infected trees (Mircetich et al., 1998). The disease can be overcome by using either ‘English’ walnut rootstocks (*J. regia*) or ‘English’ walnut cultivars growing on their own roots. Micropropagation techniques can be used to produce own-rooted ‘English’ walnut cultivars (McGranahan et al., 1988).

DT is a killed-microbial product of the fungus *Myrothecium verucaria*. The mode of action of DT is due primarily to the presence of many, relatively low-molecular-mass, water-soluble, compounds, which act synergistically (Wilson and Jackson, 2013). It has been shown to kill nematodes via contact as well as to inhibit hatching and development of eggs, cause muscle paralysis, feeding inhibition, depletion of lipids, and changes in sensory perception affecting activities such as host and mate-finding (Twomey et al., 2000, 2002; Rehberger et al., 2002). In addition to activity to nematodes, increased plant health, shoot and root weights, greening, and root proliferation have also been observed in trials by others (Spence and Lewis, 2010). Additional work has shown that it enhances microbial antagonism towards nematodes. This antagonism was associated with structural and functional changes of the rhizosphere bacterial and fungal community (Fernandez et al., 2001). DT applied at planting to strawberries in a greenhouse trial decreased populations of *Pratylenchus penetrans* and stimulated root and crown growth compared to infested controls (Pinkerton and Kitner, 2006).

NQ is an extract of *Quillaja saponaria* a tree endemic to Chile that is rich in secondary plant metabolites including saponins, glycosides, polyphenols, and tannins that are found in the cortex, leaves and flowers (Insunza et al., 2001). Aqueous extracts have been shown to have nematicidal effects against a variety of nematode species and to increase root growth (Martın and Magunacelaya, 2005).

Our trials have demonstrated that bionematicides can improve yield, growth, and vigor in walnut orchards infested with plant-parasitic nematodes. This prolongs the viable life of an orchard and reduces the frequency of pre-plant fumigations. This research contributed to the registration of these organic nematicides (OMRI certified) in California.

As we move away from traditional fumigant and nonfumigant nematicides towards natural products with different modes of action, the most effective application methods, rates, and timing, and interpretation of results become less straight forward. For example, in the trial conducted in San Joaquin County, increasing yields were associated with an increase in populations of nematodes. This could be an indication of the development of a more vigorous root system that is capable of supporting greater populations of nematodes.

For more than 30 years we have searched for a product that would replace DBCP. What we have found after years of believing that “the only good plant-parasitic nematode is a dead nematode” is that products are available that will increase yields in the presence of plant-parasitic nematodes and, may actually permit populations to increase. This opens the door to additional research on how to best utilize the new tools that we have to maximize yields for growers. It also raises questions for additional long-term research on perennial crops. Will yields continue to increase as demonstrated in these trials, will yields stabilize, or will yields crash at some point in the future? Research with bionematicides on annual crops has also shown increases in yield without a reduction in nematode populations (Westerdahl and Radewald, 2011). The current research is also a challenge to others to take another look at data they may have set aside because yields increased, but effects on nematode populations did not match expectations.
